# Supplementary figures and images for: Identification of SNPs Associated with Stress Response Traits within High Stress and Low Stress Lines of Japanese Quail
Source: Genes (Basel). 2021 Mar 12;12(3):405. doi: 10.3390/genes12030405 (PMC8000459; doi:10.3390/genes12030405)

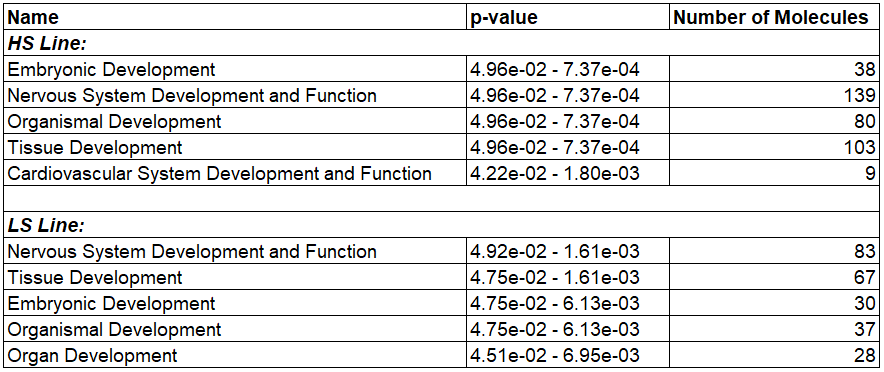

Supplement: Supplementary file 1 [file genes-12-00405-s001.zip › Supplementary Material 10_Top Physiological Functions.PNG]

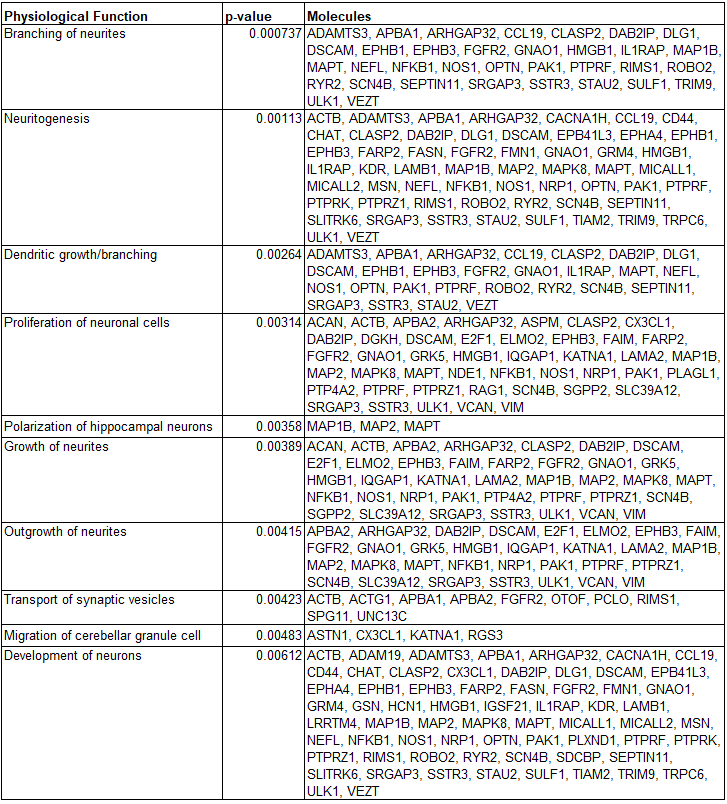

Supplement: Supplementary file 1 [file genes-12-00405-s001.zip › Supplementary Material 13_HS Line Top 10 Nervous System Functions.PNG]

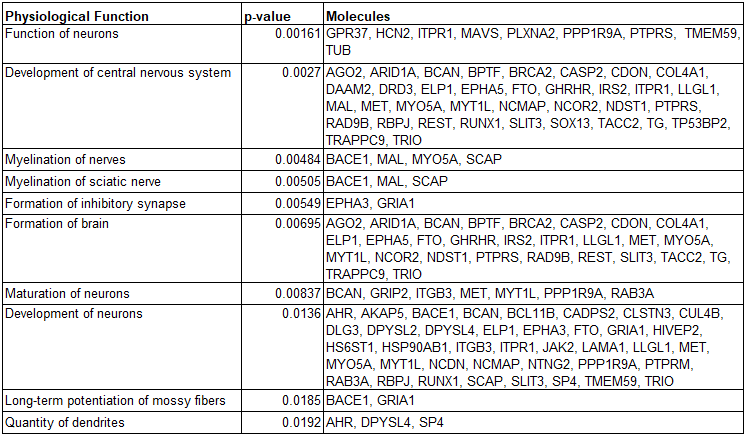

Supplement: Supplementary file 1 [file genes-12-00405-s001.zip › Supplementary Material 14_LS Line Top 10 Nervous System Functions.PNG]

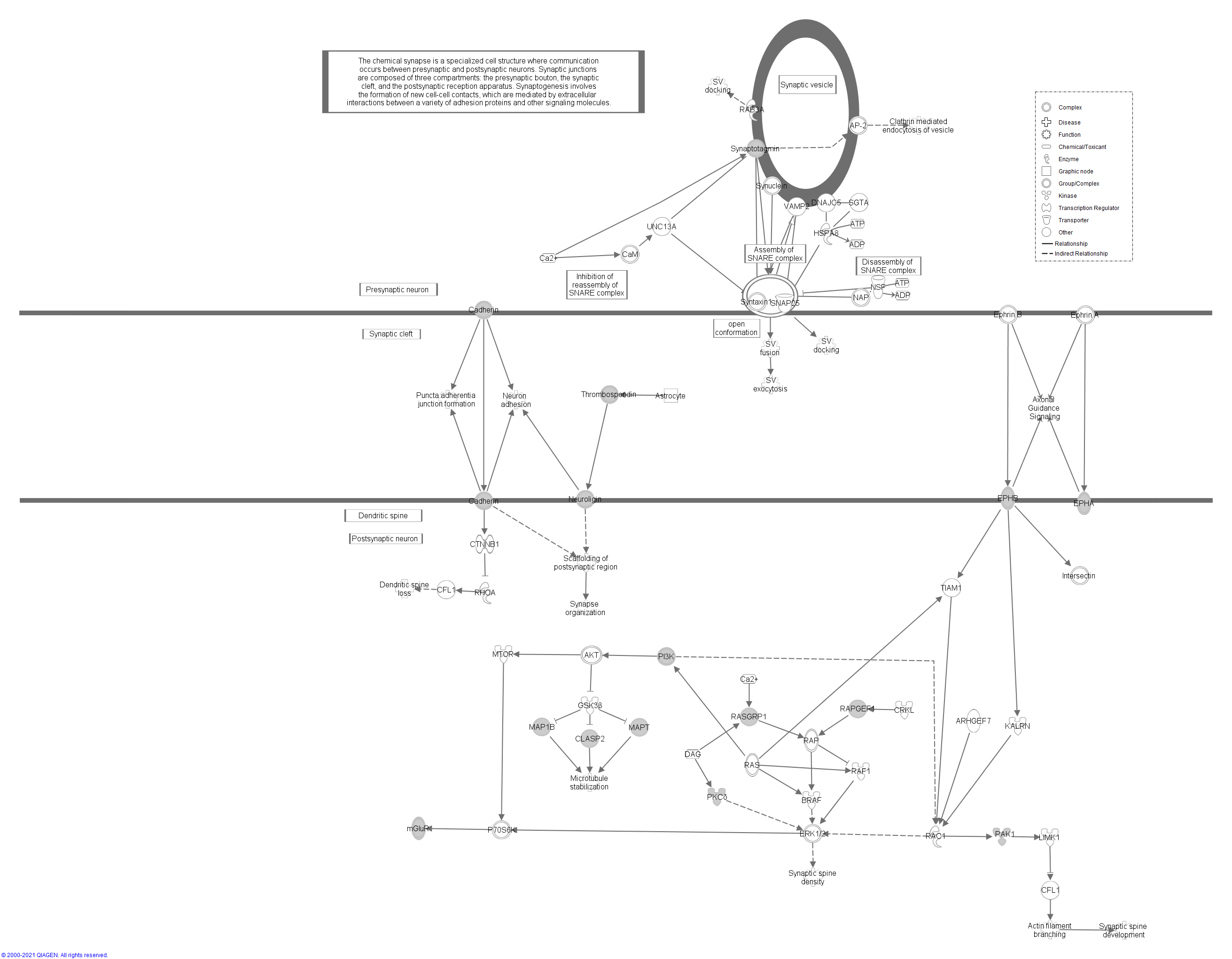

Supplement: Supplementary file 1 [file genes-12-00405-s001.zip › Supplementary Material 5_HS Line Synaptogenesis Signaling.png]

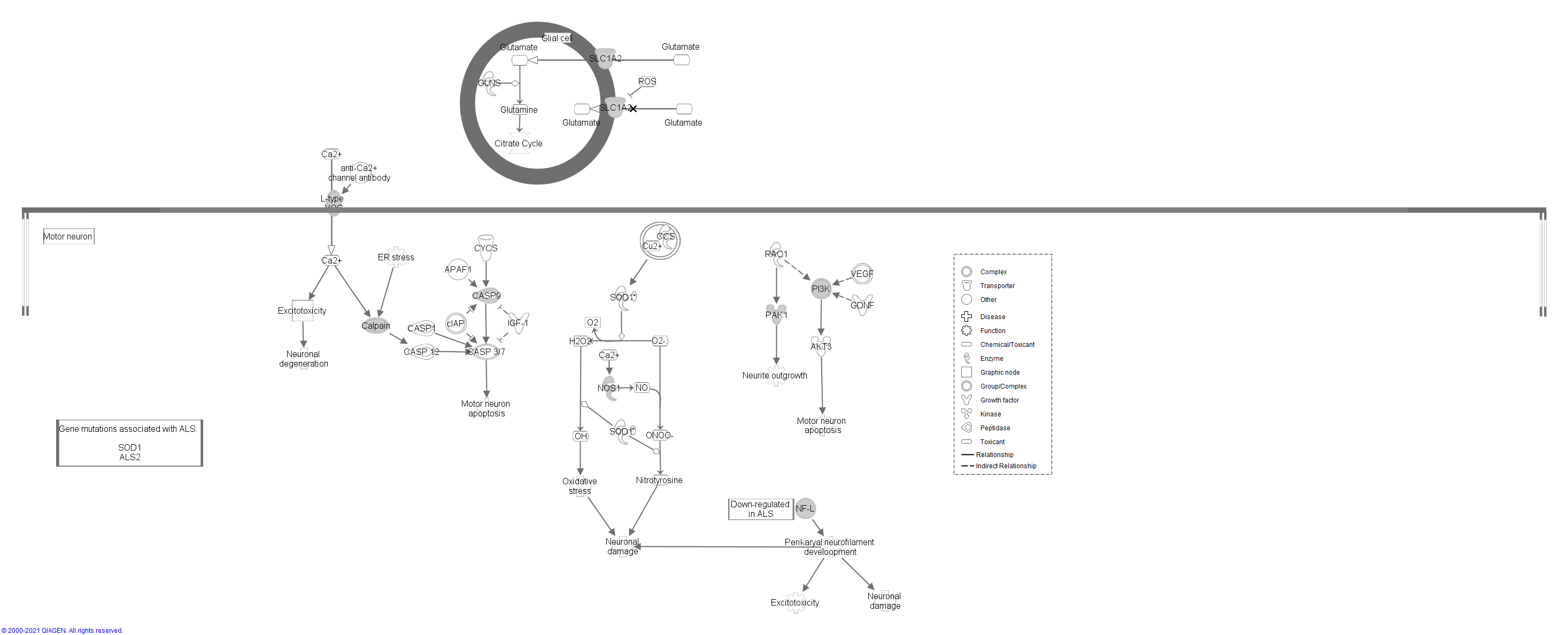

Supplement: Supplementary file 1 [file genes-12-00405-s001.zip › Supplementary Material 6_HS Line ALS Signaling.png]

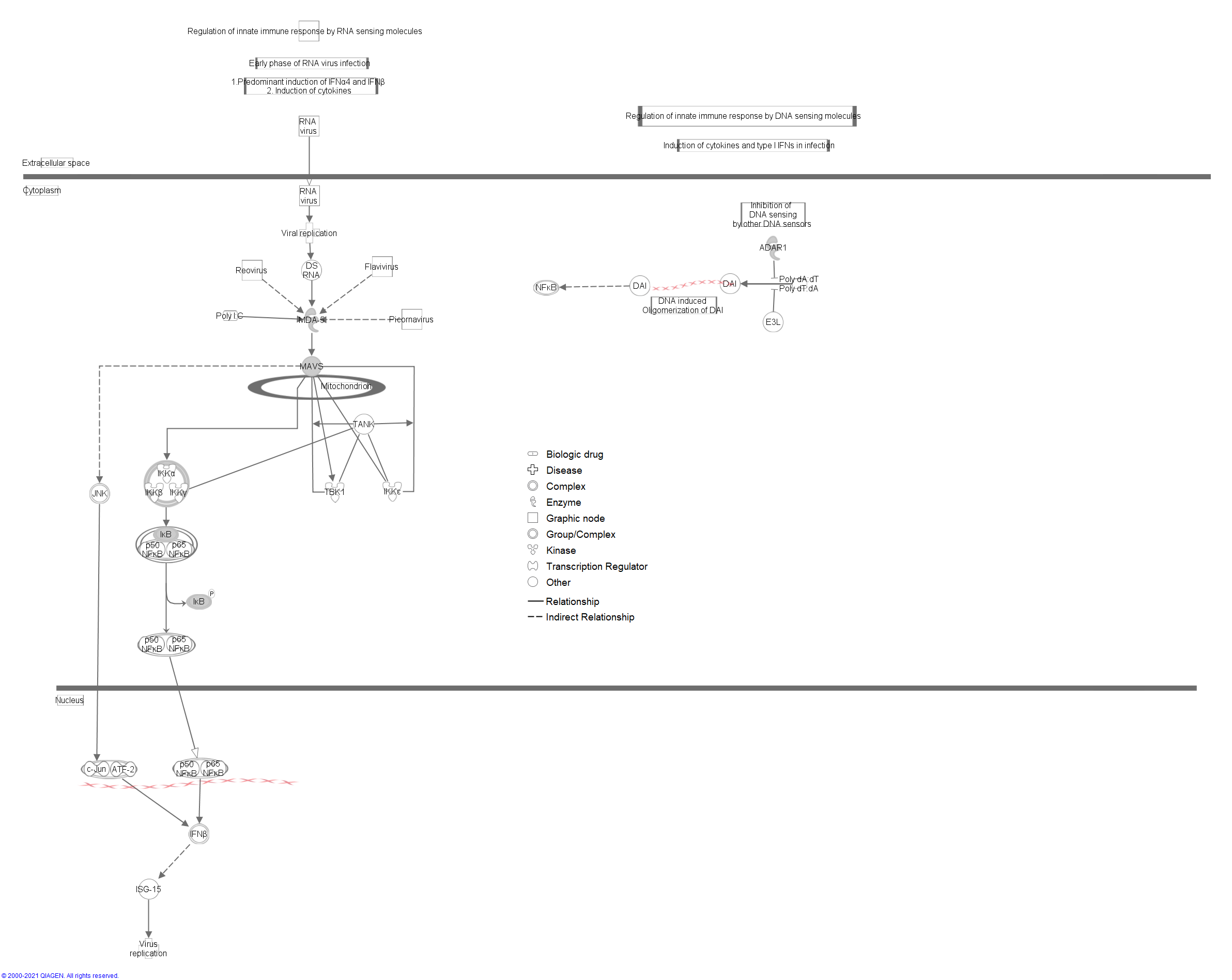

Supplement: Supplementary file 1 [file genes-12-00405-s001.zip › Supplementary Material 7_LS Line Activation of IRF.png]

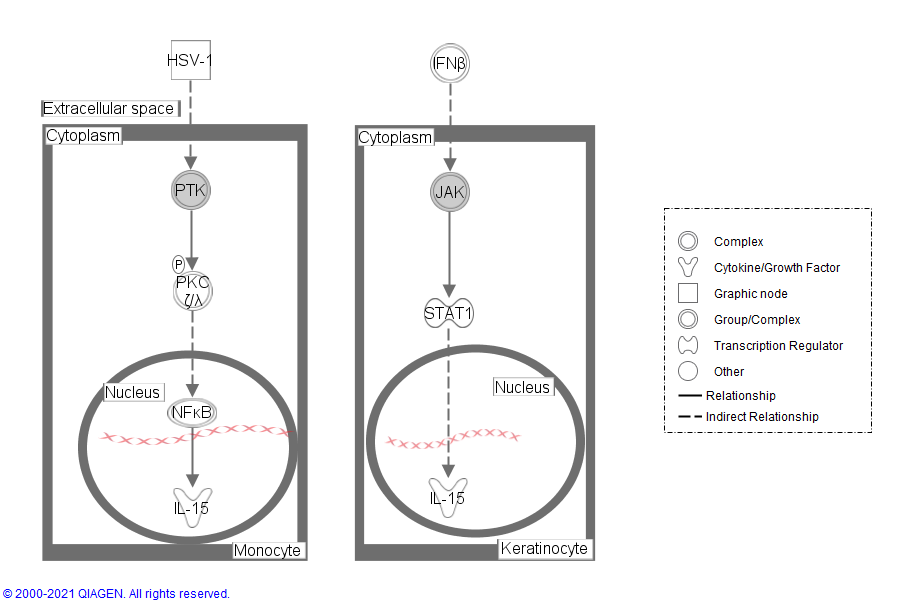

Supplement: Supplementary file 1 [file genes-12-00405-s001.zip › Supplementary Material 8_LS Line IL-15 Production.png]

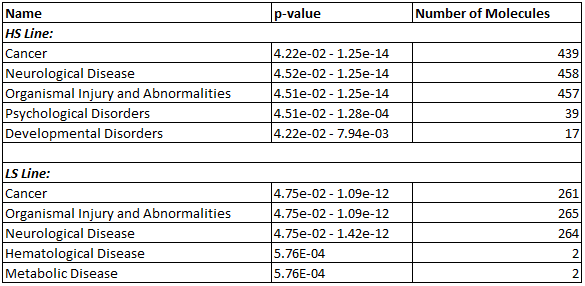

Supplement: Supplementary file 1 [file genes-12-00405-s001.zip › Supplementary Material 9_Top Diseases and Disorders Fix.PNG]
